# PAK1 expression protects cellular and behavioral defects in animal models of Parkinson’ s disease

**DOI:** 10.1186/s13041-026-01320-3

**Published:** 2026-06-11

**Authors:** Jeong Eun Kim, Kina Lee, Hee Jeong Kim, Won-Jong Oh, Hyong Kyu Kim

**Affiliations:** 1 Graduate Program in Neuroscience, Department of Medicine and Microbiology, Chungbuk National University, Cheongju, 28160 the Republic of Korea; 2 Neurovascular Unit Research Group, Korea Brain Research Institute, Daegu, 41062 the Republic of Korea

**Keywords:** PAK1, AAV, α-synuclein, Parkinson’s disease

## Abstract

Parkinson’s disease (PD) is the second most prevalent neurodegenerative disorder worldwide. Although various therapeutics have been developed, no disease-modifying treatment has been established to date. Recent studies highlight critical roles of p21-activated protein kinases (PAKs) in the pathogenesis of PD. To investigate the potential of PAK1 as a therapeutic target for PD, we examined the effects of a constitutively active form of PAK1 (PAK1-T423E; PAK1-CA) in animal models. An animal model of PD, recapitulating α-synucleinopathy was established by injecting human α-synuclein preformed fibrils into the biceps femoris of M83 A53T homozygous mice. PAK1-CA expression significantly ameliorated behavioral deficits and prevented the loss of dopaminergic neurons in the substantia nigra pars compacta (SNpc). Moreover, PAK1-CA expression increased the survival rate, although it was not significant in this model. In contrast, in the 1-methyl-4-phenyl-1,2,3,6-tetrahydropyridine (MPTP)-induced neurotoxin model, PAK1-CA expression significantly improved survival rates compared to the GFP-expressing control group. These findings suggest a potential role for constitutively active PAK1 in contributing to the neuroprotection of dopaminergic neurons, which may imply its utility as a candidate for gene therapy.

**Supplementary Information:**

The online version contains supplementary material available at 10.1186/s13041-026-01320-3.

## Main text

Parkinson’s disease is the fast growing a neurological disorder and the second most common neurodegenerative disease worldwide. The burden for treatments is steeply rising, but there is no effective disease modifying treatment to date [1, 2]. The p21-activated protein kinases (PAKs) are a serine/threonine kinase. Because of acting downstream of small GTPases such as Cdc42 and Rac1, the molecular function of PAKs is wildly ranged from cytoskeleton remodeling to gene expression [3]. Recently, it has been reported that several PAKs have critical roles in development and pathogenesis of neurodegenerative diseases, such as Alzheimer’s disease (AD), Huntington’s disease, and PD [3]. In human brains with AD, levels of PAK1 and PAK3 were significantly reduced and phosphorylated PAKs (pPAKs) were redistributed [4]. In contrast, an increase of total level of PAK1-3 at the early stages of AD is followed by a loss of soluble pPAK at the late stages [5], indicating PAKs roles in AD pathogenesis [3]. Our previous study has suggested that activity of PAK1 is involved in mesencephalic dopamine neurons in oxidative stress-induced cell death [6]. In addition, PAK4 activity is decreased in the SNpc of brain tissues with PD and the expression of a constitutively active form of PAK4 rescues the loss of dopamine neurons in the PD animal models [7]. Altogether, these suggest that PAK activity could be related with neurodegenerative diseases and its increase have protective effects on the PD pathogenesis. Accordingly, we examined the effects of a constitutively active form of PAK1 (PAK1-CA, Thr423Glu, T423E) on an animal model of PD.

**Fig. 1:**
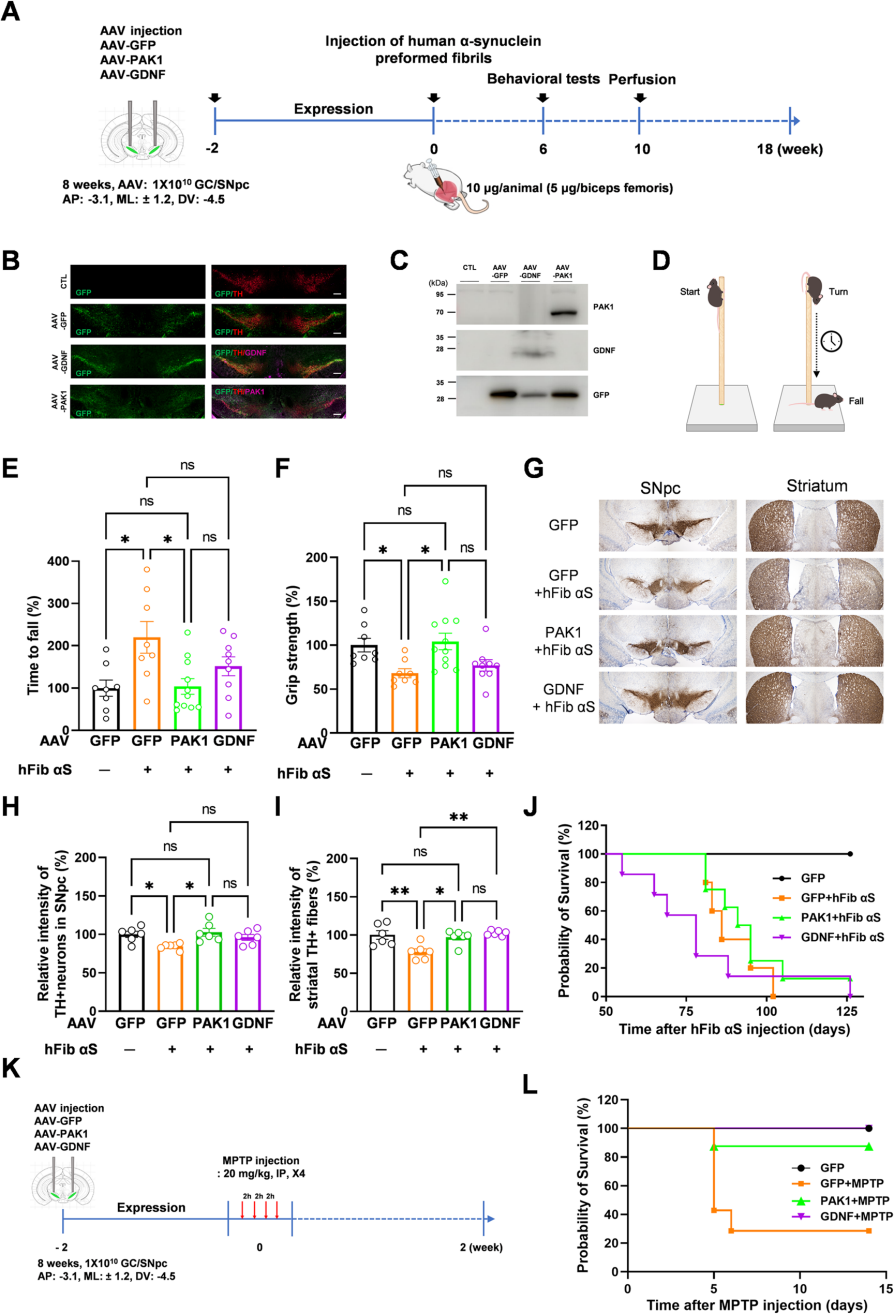
PAK1-CA expression enhances striatal dopaminergic neuron survival and improves survival in an hFib aS injection PD model. **A** A schematic for stereotaxic surgery and viral injection (AAV-hSyn-GFP, AAV-hSyn-myc-hPAK1-CA-2A-GFP, AAV-hSyn-hGDNF-myc-2A-GFP), fibrillar human α-synuclein (hFib αS) injection, and timeline for survival tests. **B** Viral infection and expression. Two weeks following viral injection, mice were perfused. Brain sections were stained with myc-antibody for PAK1 and GDNF and tyrosine hydroxylase (TH) antibody. Scale bar: mm. **C** Western blotting assay for expressed proteins by viral infections. The brain lysates including the SNpc were subjected to assays for PAK1 and GDNF using anti-myc antibody and GFP using anti-GFP antibody. **D** Scheme of the pole test **E** PAK1-CA expression recovered increased the time to fall (GFP-: 100.0% ± 19.05%, *N* = 8 animals; GFP+: 219.6% ± 37.48%, *N* = 8 animals; PAK1+: 103.8% ± 18.49%, *N* = 11 animals; GDNF+: 151.1% ± 22.33%, *N* = 9 animals; One-way ANOVA: *F*_(3,32)_ = 4.879, *P* = 0.0066; Šídák’s multiple comparisons test: * *P* < 0.05, ns: not significant). **F** PAK1-CA expression recovered the grip strength of four limbs (GFP-: 100.0% ± 7.78%, *N* = 8 animals; GFP+: 68.1% ± 4.95%, *N* = 8 animals; PAK1+: 104.1% ± 9.45%, *N* = 11 animals; GDNF+: 76.86% ± 6.51%, *N* = 9 animals; One-way ANOVA: *F*_(3,32)_ = 5.012, *P* = 0.0058; Tukey’s multiple comparisons test: * *P* < 0.05, ns: not significant). **G** PAK-CA expression recovered the loss of striatal dopamine neurons. **H** Relative intensity of TH-positive neurons in the SNpc (GFP-: 100.0% ± 3.96%, *N* = 6 hemi-midbrains from 3 animals; GFP+: 84.3% ± 1.63%, *N* = 6 hemi-midbrains from 3 animals; PAK1+: 102.7% ± 5.05%, *N* = 6 hemi-midbrains from 3 animals; GDNF+: 96.1% ± 3.90%, *N* = 6 hemi-midbrains 3 animals; One-way ANOVA: *F*_(3,20)_ = 4.470, *P* = 0.0147; Tukey’s multiple comparisons test: * *P* < 0.05, ns: not significant). **I** Relative intensity of striatal TH-positive fibers (GFP-: 100.0% ± 5.63%, *N* = 6 hemi-striata from 3 animals; GFP+: 77.9% ± 4.08%, *N* = 6 hemi-striata from 3 animals; PAK1+: 97.2% ± 3.76%, *N* = 6 hemi-striata from 3 animals; GDNF+: 102.6% ± 1.34%, *N* = 6 hemi-striata from 3 animals; One-way ANOVA: *F*_(3,20)_ = 7.843, *P* = 0.0012; Tukey’s multiple comparisons test: * *P* < 0.05, ** *P* < 0.01, ns: not significant). **J**Kaplan-Meier survival plot shows increased survival rate by PAK1-CA expression. Homozygous M83 transgenic mice were injected with the indicated AAV particles and allowed two weeks for expression. Following intramuscular injection of hFib αS, survival times were monitored (GFP-: 4 animals; GFP+: 5 animals; PAK1+: 8 animals; GDNF+: 7 animals) (Log-rank test: χ^2^ = 12.63, df = 3, *P* = 0.0055; Median survival dates: GFP+: 86 days; PAK1+: 93 days; GDNF+: 78 days). **K** A schematic for stereotaxic surgery and viral injection (AAV-GFP, AAV-PAK1, AAV-GDNF), MPTP injections, and timeline for survival tests. **L** Kaplan-Meier survival plot shows increased survival rate by PAK1-CA expression (GFP-: 3 animals; GFP+: 7 animals; PAK1+: 8 animals; GDNF+: 7 animals) (Log-rank test: χ^2^ = 12.64, df = 3, *P* = 0.0055)

The α-synuclein inclusions are hallmark of synucleinopathies, including PD, multiple system atrophy, and dementia with Lewy bodies [8]. Sacino et al. [9] proposed a convenient PD animal model, showing synchronized rapid-onset motor symptoms, α-synuclein pathology in the CNS, and the progression of α-synuclein inclusions from muscles to CNS. Accordingly, we established α-synuclein inclusions model as a PD animal model to examine the effects of PAK1 expression. The human α-synuclein (21–140 amino acids) was fibrillated and monitored with K114 fluorometry. The fibrillar human α-synuclein (hFib αS) was sonicated and examined by negative staining electron microscopy to measure the average size of fibrils (73.2 nm ± 3.1 nm) [10]. Aged 8 weeks of homozygous M83 transgenic mice expressing human Ala53Thr (A53T) mutant αS [10] were respectively injected with recombinant adeno-associated virus2/DJ -GFP (AAV-GFP), AAV2/DJ-human PAK1-CA (AAV-PAK1), or AAV2/DJ-human glial cell line-derived neurotrophic factor (AAV-GDNF), which is widely known to promote the survival of neuronal cells, to the bilateral SNpc (Fig. 1A). Two weeks following viral injection, viral expression was monitored by immunohistochemistry for PAK1, GDNF, and tyrosine hydroxylase (TH) (Fig. 1B) and western blotting assay (Fig. 1C). Subsequently, 5 µg of hFib αS was injected into each biceps femoris muscle (Fig. 1A).

Because unilateral foot drop is developed as early as 40 days post intramuscular injection of hFib αS.

 [9], six weeks following the intramuscular injection of hFib αS, motor defects of model mice were examined by pole tests and grip strength tests. The bilateral muscular injection of hFib αS was significantly increased time to fall and this increase was blocked by PAK1-CA expression, but not by GDNF expression (Fig. 1D, E). Consistently, grip strength of four limbs was decreased by the bilateral muscular injection of hFib αS. This effect was prevented by PAK-CA expression, but not by GDNF expression (Fig. 1F). The intramuscular injection of hFib αS induces both α-synuclein inclusions, and astrogliosis and microgliosis [9]. We next assessed dopaminergic neuron levels in the SNpc and striatum by immunohistochemistry using TH antibody. Ten weeks after intramuscular injection, the mice were perfused. Injection of hFib αS led to the reduction of TH-positive neurons in the SNpc. This loss was significantly attenuated by PAK1-CA expression and, although the effect did not reach statistical significance, by GDNF (Fig. 1G, H). Similarly, hFib αS injection decreased striatal TH-positive fibers, an effect that was blocked by the expression of either PAK1-CA or GDNF (Fig. 1G, I). Interestingly, while GDNF attenuated the loss of dopaminergic neurons and terminals, it failed to improve behavioral outcomes in this α-synuclein model. This observation aligns with previous reports indicating that the therapeutic efficacy of GDNF varies across different PD models [11]. In contrast, PAK1-CA expression successfully rescued both the behavioral deficits and the loss of dopaminergic neurons.

We monitored the survival of PD mouse models. While a previous study [9] reports a median survival of 77 days post-injection of human α-synuclein fibrils (hFib αS), our experiments showed median survival times of 86 days (GFP), 78 days (GDNF), and 93 days (PAK1-CA) (Fig. 1J). Although not statistically significant, PAK1-CA expression appeared to improve survival in this model. To further evaluate the neuroprotective effects of PAK1-CA, we utilized an MPTP-induced PD mouse model [12]. Eight-week-old wild-type male mice received four intraperitoneal injections of MPTP (20 mg/kg per dose) every 2 h (Fig. 1K), a protocol known to deplete striatal dopamine by 90% within 7 days [12]. Consistent with the results from the hFib αS model, PAK1-CA expression significantly increased the survival rate in the MPTP-treated mice (Fig. 1L).

PAK1 activity is associated with the level of Bcl-2 (B-cell CLL/lymphoma 2), a pro-survival protein [6] and survival of tumor cells [13]. Interestingly, Bcl-2 levels are inversely correlated with those of α-synuclein [14]. Furthermore, PAK4—a related PAK isoform—phosphorylates E3 ubiquitin ligases to promote the degradation of α-synuclein [15]. Consequently, PAK1 activation may delay the progression of α-synuclein pathology or neurotoxin-induced damage by enhancing neuronal survival within the SNpc. Collectively, our findings suggest that PAK1 activity exerts a neuroprotective effect against α-synuclein inclusions and neurotoxins, thereby improving overall survival. These data highlight the potential of PAK1 as a therapeutic target for gene therapy or as a biomarker for PD.

## Supplementary Information

Below is the link to the electronic supplementary material.

Supplementary Material 1

## Data Availability

The datasets generated and analyzed during the current study are available from the corresponding author on request.
