# Supplementary material for: PAK1 expression protects cellular and behavioral defects in animal models of Parkinson’ s disease
Source: Mol Brain. 2026 Jun 11;19:62. doi: 10.1186/s13041-026-01320-3 (PMC13479705; doi:10.1186/s13041-026-01320-3)
Supplement: Supplementary file 1 [file 13041_2026_1320_MOESM1_ESM.docx]

**Supporting information**

**Materials and Methods**

Animal use

All animal procedures were performed in accordance with the Guide for the Care and Use of Laboratory Animals and approved by the Institutional Animal Care and Use Committee of Chungbuk National University (IACUC CBNUA-24-0040-01). Mice were housed in a temperature- and humidity-controlled vivarium under a reversed 12-h light/dark cycle, with ad libitum access to food and water. To minimize bias, all behavioral tests and subsequent histological analyses were conducted by experimenters blinded to the experimental groups.

Viral expression

To construct the pAAV-hSyn-myc-hPAK1-CA (Thr423Glu, T423E)-2A-GFP plasmid, a recombinant PCR strategy was employed. The human PAK1-CA fragment was amplified from the pCMV-myc-hPAK1-CA template using the primers, Myc-NheI-S and hPAK1-2A-A. Concurrently, the GFP sequence was amplified from the pGFP-C1 vector using the primers GFP-2A-S and GFP-HindIII-A. The two resulting PCR fragments were then fused via recombinant PCR using the primers Myc-NheI-S and GFP-HindⅢ-A to generate the myc-hPAK1-CA-2A-GFP construct. This final fragment was digested with NheⅠ and HindⅢ restriction enzymes and subsequently ligated into the pAAV-hSyn-GFP (a gift from Bryan Roth, Addgene #50465) backbone to replace the original GFP sequence. To construct the pAAV-hSyn-hGDNF-myc-2A-GFP plasmid, an identical cloning strategy was employed. However, Myc-tagged human GDNF in N-terminal region was amplified form the pCDNA3.1-hGDNF-Myc-His using the primers, hGDNF-NheI-S and Myc-2A-A. All primers and restriction enzymes were purchased from Bioneer (Daejeon, South Korea) and New England Biolabs (Ipswich, MA, USA), respectively.

Table 1. Primers

| **Name** | **Sequence** | etc. |
| --- | --- | --- |
| Myc-NheI-S | 5’-CTAGCTAGCCCCACCATGGCATCAAT-3’ |  |
| hPAK1-2A-A | 5’-AGGGCCGGGATTCTCCTCCACGTCACCGCATGTTAGAA  GACTTCCTCTGCCCTCGTGATTGTTCTTTGTTGCCT-3’ |  |
| GFP-2A-S | 5’-GAGGGCAGAGGAAGTCTTCTAACATGCGGTGACGTGG  AGGAGAATCCCGGCCCTATGGTGAGCAAGGGCGAG-3’ |  |
| GFP-HindIII-A | 5’-CCCAAGCTTTTACTTGTACAGCTCGTCCA-3’ |  |
| hGDNF-NheI-S | 5’-CTAGCTAGCGCCACCATGAAGTTATGG-3’ |  |
| Myc-2A-A | 5’-GGGCCGGGATTCTCCTCCACGTCACCGCATGTTAGAAG  ACTTCCTCTGCCCTCCAGATCCTCTGAGATGA-3’ |  |

Recombinant Adeno-associated virus (AAV) particles, serotyped with DJ, were provided by the Korea Institute of Science and Technology (KIST) virus facility (Seoul, South Korea). AAV particles used in this study included AAV-hSyn-GFP (AAV-GFP, 5.18 X 10^13^ GC/ml), AAV-hSyn-hGDNF-myc-2a-GFP (AAV-GDNF, 5.22 X 10^13^ GC/ml), AAV-hSyn-myc-hPAK1-CA-2A-GFP (AAV-PAK1, 5.72 X 10^12^ GC/ml). Each viral stock was diluted in sterile saline to a final concentration of 1 X 10^10^ GC/ml. AAV particles were bilaterally injected into the substantia nigra pars compacta (SNpc) (AP, -3.1 mm; ML, ±1.2 mm; DV, -4.5 mm) of mice via stereotaxic surgery. A total volume of 2 μl was infused at a rate of 100 nl/min. Following injection, animals were allowed a two-week recovery period to ensure stable viral expression prior to subsequent experimental procedures.

Animal models

Fibrillar human α-synuclein-induced PD model

To generate fibrillar human α-synuclein (hFib αS), recombinant human α-synuclein protein (Cat# ATGPCS0092, NKMAX, Seongnam, South Korea) was concentrated to 2 mg/ml in sterile PBS. Fibrillization was induced by continuous shaking at 1,050 rpm at 37℃ for 48 hours (Thermomixer R, Eppendorf, Hamburg, Germany). The formation of fibrils was validated using K114 (Tocris Bioscience, Bristol, UK) fluorometry. The fibrils were mixed with 50 μM K114 in 100 mM glycine (pH 8.6) and incubated for 10 min at room temperature. Fluorescence was measured at excitation/emission wavelengths of 380/550 nm (cutoff = 530 nm). Prior to animal administration, the hFib αS were fragmented by sonication. Sonication was performed at 20% amplitude with 1s/1s (on/off) pulse cycle for a total of 60 seconds. The resulting suspension was then passed through a 0.45 μm membrane filter. The hFib αS was examined by negative staining electron microscopy to measure the size of fibrils (Tecnai G2 F20). The EM images were analyzed by NIH ImageJ program (Bethesda, MA, USA). Aged eight weeks of homozygous M83 transgenic mice were bilaterally injected with each AAV particles to the SNpc. Two weeks later, 5 μg of hFib αS was administered to each biceps femoris muscle (10 μg of hFib αS per animal).

MPTP-induced PD model

Aged eight weeks of male mice (C57BL/6, Samtaco Bio, Osan, Korea) were injected with each AAV particles. Two weeks after viral injection, mice were administered either 20 mg/kg of 1-Methyl-4-phenyl-1,2,3,6-tetrahydropyridine (MPTP)-hydrochloride (Cat# M0896, Sigma-Aldrich, St. Louis, MO, USA) or an equivalent volume of sterile saline intraperitoneally. A total of four injections were administered at 2-hour intervals over a single day. All MPTP handling and disposal were performed in strict accordance with institutional safety protocols for neurotoxins.

Histology and Immunohistochemistry

Animals were transcardinally perfused with 4% paraformaldehyde (Cat# P6148, Sigma-Aldrich), and brains were fixed in 4% paraformaldehyde overnight at 4℃. The fixed tissues were then dehydrated in 30% sucrose (Cat# S0389, Sigma-Aldrich) at 4℃ for 72 hours. Tissues were embedded in optimal cutting temperature (OCT) compound (Cat# 3801480, Leica Biosystems, Richmond, IL, USA) and rapidly frozen. Coronal sections (30 um thick) were obtained using a refrigerated cryostat (Cat# CM1860, Leica Biosystems). Free-floating sections were washed three times with phosphate-buffered saline and incubated 3% hydrogen peroxide for 10 minutes with orbital shaker. Following three additional PBS washes, sections were incubated in blocking solution (30% fetal bovine serum, 0.2% Triton X-100 in phosphate-buffered saline) for 1 hour at room temperature. To visualize dopaminergic neurons, sections were incubated overnight at 4℃ with a polyclonal anti-tyrosine hydroxylase (TH) antibody (1:1000; Cat# AB152, Merck Millipore, Darmstadt, Germany). After washing three times, the sections were incubated with a biotinylated secondary antibody (1:250) for 1 hour at room temperature. The signal was further amplified by incubating the sections with an avidin-biotin-peroxidase (Rabbit IgG) complex (ABC) kit (Cat# PK-4001, Vector Laboratories, Newark, CA, USA) and visualized with 3,3’-diaminobenzidine (DAB) substrate (Cat# 11718096001, Roche, Basel, Switzerland). Following signal development, slides were counterstained with thionin acetate (Cat# 861340, Sigma-Aldrich) for TH immunostaining. To quantify TH-positive neurons in the SNpc and fibers in the striatum, values were averaged across 2–3 and 3–4 sections per hemi-brain region, respectively.

For fluorescence imaging, sections were incubated with a monoclonal anti-c-myc antibody (1:1000; Clone 9E10; Cat# M4439, Merck Millipore) and a polyclonal anti-GFP antibody (1:1000; Cat# G1544, Merck Millipore) overnight at 4℃, followed by an Alexa Fluor 647-conjugated goat anti-mouse IgG (H+L) (1:250; Cat# A21235, Thermo Fisher Scientific, Waltham, MA, USA) for 1 hour at room temperature. All stained sections were mounted onto slide glass using DPX mounting solution (Cat# 06522, Sigma-Aldrich) for long-term preservation and imaging.

Western blotting assay

The midbrains isolated from mice were homogenized in a lysis buffer (150 mM NaCl, 1% IGEPAL® CA-630 and 50 mM Tris-Cl; pH 8.0) supplemented with a protease inhibitor cocktail (Cat# 11697498001, Roche, Basel, Switzerland). Tissue lysates were centrifuged at 12,000 rpm for 10 min at 4℃, and the resulting supernatants were collected. Proteins were separated by sodium dodecyl sulfate-polyacrylamide gel electrophoresis (SDS-PAGE) using 8–12 % gels and subsequently transferred to 0.45 μm polyvinylidene difluoride (PVDF; Cat# IPVH00010, Merck Millipore) membranes. The membranes were pre-blocked with 5% skimmed milk in phosphate-buffered saline, and incubated with a monoclonal anti-c-myc antibody (1:2000; Clone 9E10; Cat# M4439, Merck Millipore) or polyclonal anti-GFP antibody (1:2000; Cat# G1544, Merck Millipore) followed by incubation with a peroxidase (HRP)-conjugated secondary antibodies (anti-mouse: #115-035-146, anti-rabbit: 111-035-144, Jackson ImmunoResearch Lab, West Grove, PA, USA). The signals were visualized using chemiluminescence HRP substrate (Cat# WBKLS0100, Merck Millipore) and imaged with a chemiluminescence detection system (ImageQuant 800, Cytiva, Marlborough, MA, USA).

Behavior test

Grip strength test

To assess muscular strength and neuromuscular function, the grip strength test was conducted using a digital grip strength meter (Biosep, Vitrolles, France) six weeks post-intramuscular injection of hFib αS. Prior to the test, mice were allowed to habituate to the experimental environment in their home cages for at least 30 minutes. The experimenter held the mouse by the base of its tail and allowed both the forepaws and hind paws to grasp the metal grid. Once the mouse firmly gripped the grid with all four paws, it was pulled horizontally away from the sensor with steady, gentle force until its grip was released. This procedure was repeated three times per animal and the values were averaged.

Pole test

To assess motor coordination, the pole test was performed six weeks following the intramuscular injection of hFib αS. Mice were placed on top of a vertical wooden pole (1 cm in diameter, 50 cm in height) with their heads facing upward. The time required for the mouse to orient its body downward and descend to the floor (time to fall) was recorded. Each animal performed three trials, and the average time was used for statistical analysis.

Statistical analysis

Data obtained were subjected to normality tests. If they passed the normality test, they were included in the parametric analysis, otherwise non-parametric analysis. Statistical significance among groups was measured using one-way analysis of variance (ANOVA) for parametric data. Statistical significance between groups was measured using *post hoc* multiple comparison tests (Šídák’s multiple comparisons test or Tukey’s multiple comparisons test). Data are presented as mean ± standard error of mean (SEM). Analyses with *P <* 0.05 were considered as significant. To measure significance among the survival curves, Log-rank tests were performed (GraphPad Prism ver. 9.0, La Jolla, CA, USA).

**Supplementary Data**

Sup Fig.1 EM analysis of fibrillar human α-synuclein (hFib αS). (A) Representative images of negative staining electron microscopy of hFib αS. Before and after sonication, hFib αS was visualized. (B) The average size of preformed fibrils of α-synuclein were measured (n = 194).

Sup Fig.2 Effects of PAK1 activation on α-synuclein aggregates. PAK1 activation is hypothesized to mitigate α-synuclein aggregation through two distinct pathways. First, PAK1 stabilizes and phosphorylates Bcl-2 [1], a pro-survival factor whose levels are inversely correlated with α-synuclein [2]. This suggests that PAK1 activation could stimulate survival signaling to promote aggregate clearance. Second, by analogy to its isoform PAK4—which drives α-synuclein degradation through direct interaction with the NEDD4-1 E3 ligase [3]—PAK1 may similarly enhance the clearance of these aggregates via the ubiquitin-proteasome system.

1.	Kim H, Oh JY, Choi SL, Nam YJ, Jo A, Kwon A, Shin EY, Kim EG, Kim HK. Down-regulation of p21-activated serine/threonine kinase 1 is involved in loss of mesencephalic dopamine neurons. *Mol Brain.* 2016, 9(1):45. doi:10.1186/s13041-016-0230-6.

2.	Yuan Y, Jin J, Yang B, Zhang W, Hu J, Zhang Y, Chen NH. Overexpressed alpha-synuclein regulated the nuclear factor-kappaB signal pathway. *Cell Mol Neurobiol.* 2008, 28(1):21-33. doi:10.1007/s10571-007-9185-6.

3.	Won SY, Park JJ, You ST, Hyeun JA, Kim HK, Jin BK, McLean C, Shin EY, Kim EG. p21-activated kinase 4 controls the aggregation of alpha-synuclein by reducing the monomeric and aggregated forms of alpha-synuclein: involvement of the E3 ubiquitin ligase NEDD4-1. *Cell Death Dis.* 2022, 13(6):575. doi:10.1038/s41419-022-05030-1.
